# Estimation of Real-World Vaccination Effectiveness of mRNA COVID-19 Vaccines against Delta and Omicron Variants in Japan

**DOI:** 10.3390/vaccines10030430

**Published:** 2022-03-11

**Authors:** Sachiko Kodera, Essam A. Rashed, Akimasa Hirata

**Affiliations:** 1 Department of Electrical and Mechanical Engineering, Nagoya Institute of Technology, Nagoya 466-8555, Japan; kodera@nitech.ac.jp (S.K.); essam.rashed@nitech.ac.jp (E.A.R.); 2 Department of Mathematics, Faculty of Science, Suez Canal University, Ismailia 41522, Egypt; 3 Center of Biomedical Physics and Information Technology, Nagoya Institute of Technology, Nagoya 466-8555, Japan

**Keywords:** COVID-19, vaccination strategy, waning immunity, Japan

## Abstract

A resurgence of COVID-19-positive cases has been observed in many countries in the latter half of 2021. The primary reasons for this resurgence are the waning immunity of vaccination after the second dose of vaccination and the changes in public behavior due to temporal convergence. The vaccination effectiveness for the omicron and delta variants has been reported from some countries, but it is still unclear for several other regions worldwide. Here, we numerically derived the effectiveness of vaccination for infection protection in individuals and populations against viral variants for the entire Japanese population (126 million). The waning immunity of vaccination for the delta variant of Japanese individuals was 93.8% (95% CI: 93.1–94.6%) among individuals <65 years of age and 95.0% (95% CI: 95.6–96.9%) among individuals ≥65 years of age. We found that waning immunity of vaccination in individuals >65 years of age was lower than in those <65 years of age, which may be attributable to human behavior and a higher vaccination rate among individuals >65 years of age. From the reported data of 25,187 positive cases with confirmed omicron variant in Tokyo in January 2022, the effectiveness of vaccination was also estimated at 62.1% (95% CI: 48–66%) compared to that of the delta variant. Derived effectiveness of vaccination would be useful to discuss the vaccination strategy for the booster shot, as well as the status of herd immunity.

## 1. Introduction

The emergence of COVID-19 has been a significant cause of mortality worldwide, accounting for more than 5.6 million deaths [1]. In mid-2021, the COVID-19 pandemic became temporarily controlled in some European countries, supported by high vaccination rates [2]. Although mass immunization has been achieved [3,4], COVID-19 resurgence was observed in several countries in the latter half of 2021.

With respect to vaccination rates, one of the leading countries is Israel, where a relatively high number of daily positive cases (DPCs) was reported in August 2021, at a time when the vaccination rate was above 68% [5]. This upsurge was partially attributable to the high infectivity of the delta variant [6] and the waning immunity of vaccination, which is caused by the reduction of antibodies over time, especially for those who were vaccinated very early [7]. After a third shot, the number of new DPCs decreased again, whereas there was a resurgence of COVID-19 positivity in other European and American countries [8].

Vaccination efficacy and effectiveness are often used as measures of a vaccine. Vaccination efficiency is derived under ideal or laboratory conditions, which does not always translate to effectiveness. Therefore, efficacy trials can overestimate a vaccine’s impact in practice, which is defined as vaccination effectiveness (observatory study).

Vaccination effectiveness for infection protection in different countries is variable in terms of quantity (vaccination ratio) and quality (different types of vaccines). In most European countries, there has been a resurgence of COVID-19 positivity, even in areas with high vaccination rates (60%–70%). In Japan, however, the number of new DPCs has been kept at a low level (less than a few cases per million) after the second vaccination shot, and 80% of the population has been fully vaccinated.

Among the vaccinated population in Japan, approximately 90% of individuals were vaccinated with the Pfizer BNT162b2; the rest received the mRNA-1273 Moderna COVID-19 vaccine. The schedule of the first and second vaccinations was almost harmonized throughout the country. Starting at the end of August, the number of new DPCs decreased; it remained at levels below 300 cases from November to mid-December 2021 across a population of over 120 million [9]. Similar to other countries, the waning immunity of the vaccination was a concern. As the viral omicron variant emerged, this triggered the essential need for a third booster shot. By the new year holiday season (January 2022), resurgence spiked in Japan.

One of the key metrics used to express the waning immunity of vaccination is the individual effectiveness of vaccination (IEV) [10], which is also needed for vaccination planning [11] as well as the projection of new DPCs [12]. Substantial efforts were made to derive this metric [13,14,15]. However, investigations of IEV for the omicron variant are still limited [16,17].

Here, we numerically evaluate the waning effect of vaccination in infection prevention among the Japanese population. The main goal of this study was to numerically estimate, with limited data, the waning immunity of vaccination for the whole Japanese population (126 million). These results are important to validate the potential risk triggered by weak vaccination effectiveness that may be caused by waning effect.

## 2. Materials and Methods

### 2.1. Data

The waning immunity of vaccination among the Japanese population was estimated based on data provided by the Ministry of Health, Labor, and Welfare and the Government Chief Information Officers’ (CIO) Portal [18]. The first dataset [19] includes the number of unvaccinated, partially vaccinated, and fully vaccinated individuals, and the number of infected individuals in each category. These data were provided weekly for two age categories (>65 and <65 years of age) from 1 September to 4 October 2021, and for 11 age categories (0–11, 12–19, 20–29, 30–39, 40–49, 50–59, 60–64, 65–69, 70–79, 80–89, and >90 years of age) from 4 October to 28 November 2021. For consistency, the 11 age categories were merged into 2 age categories. A summary of the original data is listed in two categories (the threshold is 65 years) in Table 1. During this period, the delta variant was dominant. Similarly, the data for the period when the Omicron variant was predominant (>87%) are listed in Table 2, from 11–20 January 2022 in Tokyo [20]. Note that during this period, the overall percentage of the omicron variant was low in the suburbs. Thus, the data were focused on Tokyo. The overall age categories are not given for the data of Tokyo. Subjects without information regarding the vaccination were excluded from this study (approximately 30%).

**Figure 1:**
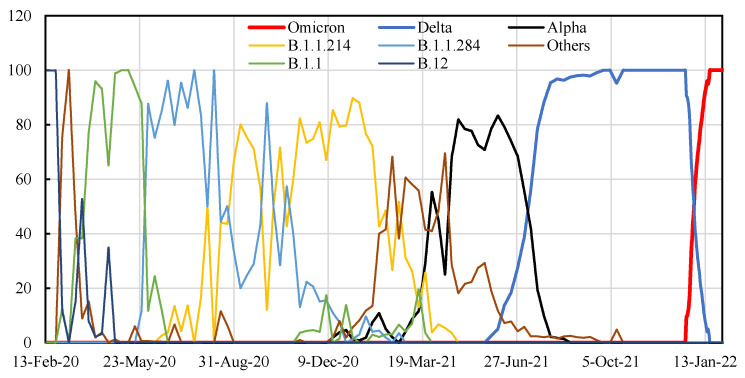
Time course of SARS-CoV-2 sequences by variants in Japan.

Vaccination rates were obtained from the Government CIOs’ Portal, Japan. These data included the daily number of vaccinated people in two age categories (>65 and <65 years of age). During the vaccination campaign, delta was the dominant variant among individuals <65 years of age, whereas the alpha variant was partly relevant for people >65 years of age (Figure 1) [9].

### 2.2. Estimation of Waning Immunity against the Delta Variant

The association between the rate of confirmed positive cases and the number of weeks after vaccination provides a measure of waning immunity. Vaccination in Japan began around March 2021 for elderly individuals and June 2021 for nonmedical workers, almost uniformly across the country. Thus, our discussion focuses on the waning effect of protection against the delta variant, which was the predominant COVID-19 variant during the study period (>80%) [9]. Waning immunity is assumed to be linear with time.

(1) $$e_{t}\left(i\right)=\{\begin{array}{c} a_{t}\cdot i/K\;\left(j\leq K\right)\\ a_{t}-s\left(i-K\right)\;\left(j>K\right) \end{array}$$

where *e_t_*(*i*) is the IEV on *i* days after inoculation for *t* dose (=1 or 2); parameters *a* and *s* are adjusted to reach a peak *K* days after inoculation, then linearly decrease. The IEV of the first shot was assumed to be constant after 14 days due to a lack of data.

### 2.3. Population Effectiveness of Vaccination

The population effectiveness of vaccination (PEV) is required to estimate the effective unprotected population from infection. The PEV *E* was assumed to be as follows:

(2) $$E\left(d\right)=\overset{T}{\underset{t=1}{\sum }}\overset{d}{\underset{i=0}{\sum }}\left(N_{t}\left(d-i\right)\cdot e_{t}\left(i\right)\right)/P$$

where *d* is the day index and *N_t_* denotes the number of people who were newly administered a vaccination shot *t* (=1 or 2). *P* is the deemed population, expressed as the summation of the population of the entire prefecture and the cumulative number of second doses, considering the waning immunity after time elapsed since the vaccination.

The daily number of people who have insufficient vaccine protection can be estimated by multiplying the total population by (1 − *E*(*d*)), calculated in Equation (2). The optimal parameters *a_t_* and *s* in Equation (1) were then determined by comparing the number of positive cases per 100,000 total population and per 100,000 individuals with insufficient protection against infection in two age categories (<65 and ≥65 years of age; Table 1).

The evaluation period was set from 1 September to 28 November 2021 for the delta variant and from 11 to 20 January 2022 for the omicron variant. These parameters for the delta variant were first set such that the slope of their regression lines matched each other. Then, the IEV for the omicron variant was reduced, assuming that the waning effect of the omicron and delta variants was linear. Note that the ratio of people who had immunity by infection was marginal because the number of people infected with the delta and omicron variants until 17 December 2021 was less than 1.5% of the total population. The PEV was calculated using an in-house python code; simultaneously, optimal parameters *a_t_* and *s* in Equation (1) were computed for reported data.

## 3. Results

**Figure 2:**
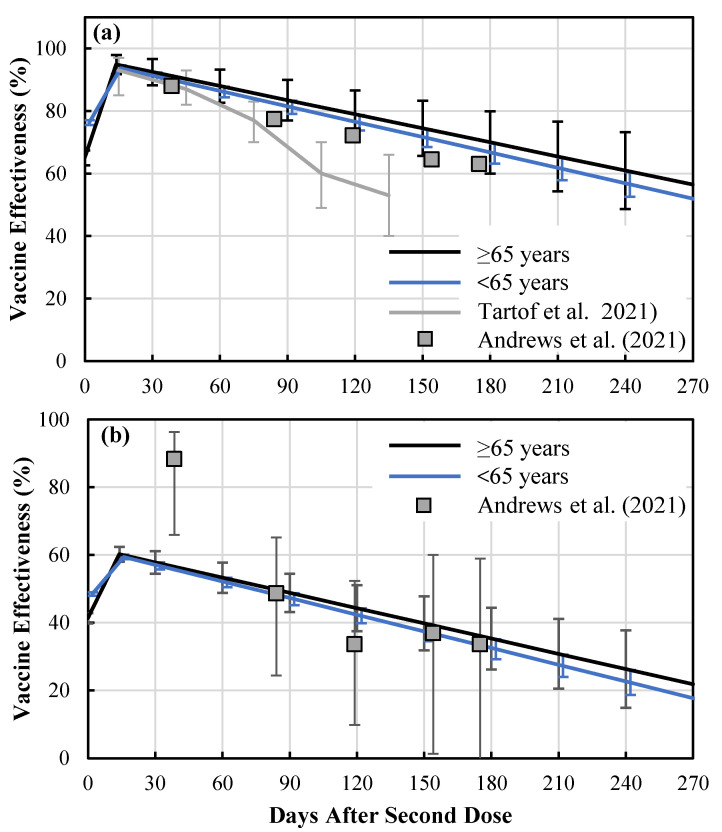
Waning immunity of vaccination in Japan, for individuals over and under 65 years of age, with respect to protection against symptomatic infection by (**a**) delta and (**b**) omicron variants derived for Japanese population. Bars represent the standard deviation.

**Figure 3:**
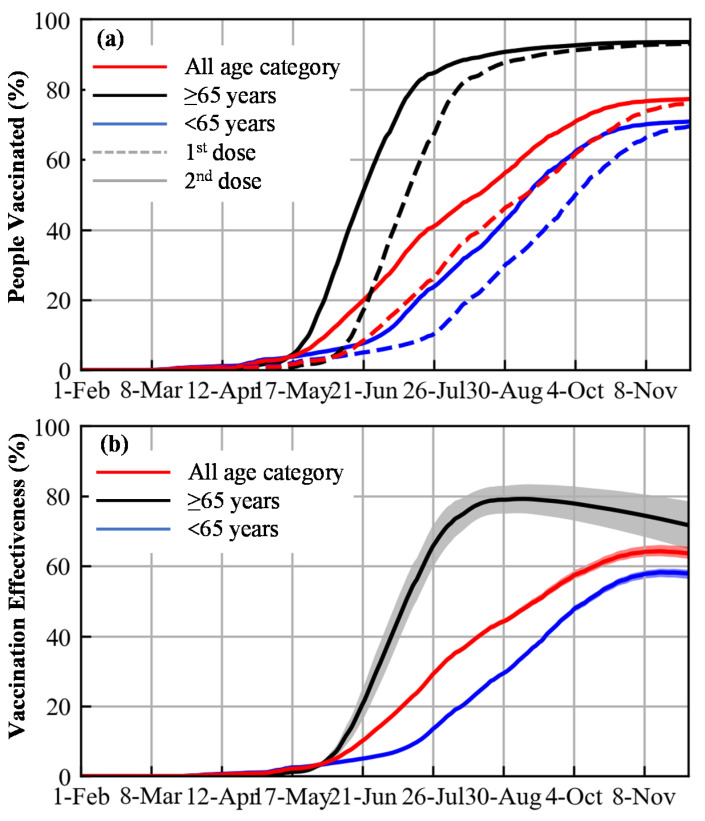
(**a**) Rate of vaccination and (**b**) population effectiveness of vaccination (PEV) for all ages, and those under and over 65 years of age for delta variant.

Figure 2 shows the waning immunity of vaccination among the Japanese population over and less than 65 years old. As shown in Figure 2a, waning immunity was almost linear for individuals both over and under 65 years of age. Due to the brief intervals for the population younger than 65 years old (approximately 2.5 months, as shown in Figure 3), the difference in waning immunity was small, whereas an IEV of approximately 95% was estimated just after the second shot. The coefficient of determination was 0.9984 (*p* < 0.0001) and 0.9927 (*p* < 0.0001) for individuals under and over 65 years of age, respectively. Based on the ratio between percentage of positive cases in unvaccinated and fully vaccinated individuals for delta and omicron variants (Table 2), the waning immunity of the omicron variant was estimated as 64.5% (95% CI: 50%–68%) of that for the delta variant using the least squares method. In the following discussion, IEV for protection against infection with omicron was assumed as shown in Figure 2b. The parameters in Equation (1) are shown in Table 3.

Figure 3 shows the vaccination rate and PEV based on statistics from the Japanese population. As shown in Figure 3a, the rate of vaccination was higher among individuals ≥65 years of age than those <65 years of age. This resulted in a higher PEV for individuals ≥65 years of age (Figure 3b).

## 4. Discussion

In this study, we numerically derived the IEV for the delta variant for the entire Japanese population of 126 million. The feature of our approach is that with limited observational data, the IEV can be estimated with simple computation. In addition, for inhomogeneous data for the population, the IEV was numerically estimated. Before this study, higher levels of protection against COVID-19 infection of the delta variant were reported in a cohort study [21]; the IEV against the delta-variant infections following full vaccination was 93% (95% CI: 85%–97%) in the first month after vaccination, but declined to 53% (95% CI: 39%–65%) after four months. According to a meta-analysis of a systematic review (11 study groups) [22], the *a*_1_ and *a*_2_ parameters for the delta variant were estimated as 60.5% and 75.6%, respectively.

The IEV obtained here was higher than that of the real-world IEV reported in other countries [23]. One potential reason for this discrepancy could be our behavior including mask wearing, which remained at approximately 90% even after full vaccination [24]. This may suggest that our comparison offers a more appropriate insight for vaccinated and unvaccinated populations in comparison to results reported in other countries. Note that mask wearing is more common in the unvaccinated populations in most countries. Another reason for this discrepancy is the PEV for individuals ≥65 years of age, which was higher than that for individuals <65 years of age (see Figure 2b). This different tendency can be hypothesized in terms of community [25]: the percentage of infected people ≥65 years would be lower due to a higher vaccination rate. Another potential bias is the population younger than 12 years, for whom the vaccination was not yet licensed. The daily number of vaccinated people are available only in two age categories (≥65 and <65 years of age) for the entire population, and thus could not be evaluated.

The IEV of the omicron variant was reduced to 64.5% of that of the delta variant, which is slightly higher than 55.0% (95% CI: 44.0–65.9%) in the U.K. [16]. Another study suggested a very low IEV in Canada [17]. Further studies are needed to clarify the difference, as well as the IEV for hospitalization [20].

A limitation of the current derivation is that we adjusted vaccination effectiveness for two age categories due to a lack of detailed data for the entire Japanese population. For the omicron variant, available data were limited to Tokyo (25,187 positive cases) in addition to a relatively short time period as it became the dominant pandemic variant starting in January 2022.

In summary, the IEV of infection protection for mRNA COVID-19 vaccines was numerically estimated using limited information for the entire population of Japan, whereas the tendency obtained here corresponds to those obtained in previous studies reported in other countries. Such computational estimation would be especially useful for vaccination planning in the early stage of viral spread.

## Figures and Tables

**Table 1:** Number of total new positive cases and cases among unvaccinated individuals. Data are summarized based on reports from the COVID-19 advisory board of the Ministry of Health, Labor, and Welfare (https://www.mhlw.go.jp/stf/seisakunitsuite/bunya/0000121431_00294.html, accessed on 3 March 2022). Individuals (**a**) over and (**b**) under 65 years of age.

(a)			
Period	Total Number of Positive Cases	Positive Cases in Unvaccinated Individuals	Number of Unvaccinated Individuals
1 September–3 September 2021	2465	1194	3,585,777
8 September–10 September 2021	1857	883	3,695,287
15 September–17 September 2021	1177	502	3,536,959
27 September–3 October 2021	1002	422	3,345,111
4 October–10 October 2021	757	316	2,799,978
11 October–17 October 2021	476	156	2,705,981
15 October–21 October 2021	416	143	2,650,175
18 October–24 October 2021	377	141	2,562,327
1 November–7 November 2021	228	64	2,498,539
8 November–14 November 2021	186	55	2,453,056
15 November–21 November 2021	194	34	2,420,586
22 November–28 November 2021	106	18	2,406,909
**(b)**			
**Period**	**Total Number of Positive Cases**	**Positive Cases in Unvaccinated Individuals**	**Number of Unvaccinated Individuals**
1 September–3 September 2021	39,580	32,098	52,133,029
8 September–10 September 2021	22,699	17,854	45,993,050
15 September–17 September 2021	13,087	10,070	41,176,559
27 September–3 October 2021	8326	6211	34,158,814
4 October–10 October 2021	5021	3636	34,117,240
11 October–17 October 2021	3069	2128	31,961,836
15 October–21 October 2021	2171	1457	30,738,527
18 October–24 October 2021	1836	1200	29,270,194
1 November–7 November 2021	1235	803	27,957,639
8 November–14 November 2021	1098	702	27,276,507
15 November–21 November 2021	769	458	26,881,777
22 November–28 November 2021	596	347	26,595,118

**Table 2:** Number of new positive cases and cases among unvaccinated individuals. Data are summarized based on reports from the Disaster Prevention Information (https://www.bousai.metro.tokyo.lg.jp/taisaku/saigai/index.html, accessed on 3 March 2022).

Date	Positive Cases of Unvaccinated Individuals	Positive Cases of Fully Vaccinated Individuals	Total Number of Unvaccinated Individuals	Total Number of Fully Vaccinated Individuals
11 January 2022	474	1071	3,541,187	10,302,142
12 January 2022	695	1549	3,540,034	10,303,295
13 January 2022	886	1970	3,538,869	10,304,460
14 January 2022	1035	2224	3,537,066	10,306,263
15 January 2022	960	1998	3,533,537	10,309,792
16 January 2022	886	1970	3,531,891	10,311,438
17 January 2022	1279	2452	3,530,896	10,312,433
18 January 2022	1836	3408	3,530,134	10,313,195
19 January 2022	2189	4024	3,529,561	10,313,768
20 January 2022	2441	4521	3,528,934	10,314,395

**Table 3:** Parameters for the individual effectiveness of vaccination (IEV) used in Equation (1) for delta and omicron variants.

	Delta		Omicron	
	Mean	95% CI	Mean	95% CI
	All-age category			
*a*_1_	74.9	(70.8–78.9)	48.3	(35.6–53.7)
*a*_2_	96.2	(95.6–96.9)	62.1	(48.0–66.0)
*s*	0.149	(0.132–0.165)	0.149	(0.132–0.165)
	Under 65 years of age			
*a*_1_	76.3	(73.9–78.7)	49.2	(37.1–53.6)
*a*_2_	93.8	(93.1–94.6)	60.5	(46.7–64.4)
*s*	0.164	(0.152–0.177)	0.164	(0.152–0.177)
	Over 65 years of age			
*a*_1_	65.0	(70.8–78.9)	41.9	(22.9–57.5)
*a*_2_	95.0	(95.6–96.9)	61.3	(46.5–66.3)
*s*	0.150	(0.111–0.189)	0.150	(0.111–0.189)

## Data Availability

Not applicable.
